# Implementation experiences and insights from the scale‐up of an HIV assisted partner notification intervention in Central Asia

**DOI:** 10.1002/jia2.25313

**Published:** 2019-07-19

**Authors:** Kristen M Little, Maxim Kan, Olga Samoylova, Altynai Rsaldinova, Daniyar Saliev, Faridun Ishokov, Robert Gray, Nina S Hasen

**Affiliations:** ^1^ Population Services International (PSI)/Washington Washington DC USA; ^2^ PSI/Central Asia Almaty Kazakhstan; ^3^ PSI/Kazakhstan Almaty Kazakhstan; ^4^ PSI/Kyrgyz Republic Bishkek Kyrgyz Republic; ^5^ PSI/Tajikistan Dushanbe Tajikistan

**Keywords:** HIV/AIDS, Central Asia, index testing, assisted partner notification, people who inject drugs

## Abstract

**Introduction:**

WHO recommends assisted partner notification (APN) for people living with HIV (PLHIV). These services have not been widely scaled in Central Asia. We describe the results from an APN intervention implemented within a programme focused on PLHIV and people who inject drugs in Kazakhstan, the Kyrgyz Republic and Tajikistan.

**Methods:**

Routine data from index cases and their partners were analysed from equal‐length periods before and after APN launch. Prior to APN index cases could recruit partners using passive referral, and under APN, had their choice of passive referral or APN (provider, contract or dual‐referral). We compared the demographic characteristics of index cases and their sexual/injecting partners from the pre‐APN and APN periods, described the number/proportion of HIV cases found (positivity rate) and evaluated predictors of HIV infection among partners using logistic regression.

**Results:**

Under APN 2676 PLHIV served as index cases and recruited 3735 partners for testing, compared to 4418 index cases and 2240 partners during the pre‐APN period. A total of 322 (8.6%) partners were rapid test positive during APN versus 161 (7.2%, *p* = 0.048) before APN. Women represented 38% of APN index cases (vs. 42% pre‐APN), 52% of partners tested (vs. 50% pre‐APN) and 56% of all PLHIV identified (vs. 63% pre‐APN). Compared to the pre‐APN period, the number of partners tested per index case recruited increased (0.5 to 1.4, *p* < 0.001) and the number of index cases needed to find one HIV‐positive partner decreased significantly (27.4 to 8.3, *p* < 0.001) under APN.

**Conclusions:**

APN was feasibly integrated within a people who inject drugs and PLHIV‐focused HIV programme, and was acceptable to high‐risk populations in Central Asia. Under APN, large numbers of sexual and injecting partners of PLHIV – including women and non‐marital partners – were tested while maintaining high positivity rates. Relative to the pre‐APN period, APN approximately tripled the number of partners recruited per index case and reduced the number of index cases needed to find a positive partner by >3 times.

## 1 Introduction

The HIV epidemics in Central Asia are highly concentrated, disproportionately impacting people who inject drugs (PWID) and their sexual partners. There are an estimated 168,600 PWID in Kazakhstan, the Kyrgyz Republic and Tajikistan, among whom HIV prevalence estimates range from 9.3% to 13.5%, compared to 0.13% to 0.19% among the general population 1, 2, 3. Obstacles to HIV epidemic control in Central Asia are numerous, including laws and policies that discriminate against key populations (KP), stigma and marginalization that limit access to HIV services, and minimal epidemiological data to inform programme design and targeting 4.

While most HIV transmission in the region is attributable to injection drug use, there are concerns that sexual transmission is contributing to an increasing share of new infections 5, 6, 7. To address the high risk of HIV acquisition among sexual partners of people living with HIV (PLHIV), the World Health Organization (WHO) recommends the implementation of partner notification services, defined as a “voluntary process where trained health workers … ask people diagnosed with HIV about their sexual or drug injecting partners, and with the consent of the HIV‐positive client, offer these partners voluntary HIV testing” 8. While these services can be passive or active, assisted partner notification (APN) services – including provider, contract or dual‐referral options – have been shown to improve the uptake and positivity rates 9, 10, 11, 12, and the WHO recommends that multiple APN options be offered 8 to meet clients’ diverse needs.

Despite the benefits, APN services have not been scaled in Central Asia. Additionally, data are limited on the acceptability and feasibility of APN in the context of PWID‐focused HIV programmes 10, 13, such as this one implemented under the United States Agency for International Development (USAID)‐funded Flagship Project in Kazakhstan, the Kyrgyz Republic and Tajikistan. Recognizing the transmission risks faced by the partners of their PLHIV clients, the project integrated APN into its HIV case‐finding and management programming. Here, we compare case‐finding outcomes, and positivity rates before and after APN implementation, and lessons learned.

## 2 Methods

### 2.1 Programme population

We analysed routine data from non‐governmental organizations (NGOs) in nine sub‐national units across Kazakhstan, the Kyrgyz Republic and Tajikistan implementing Flagship, which provided HIV case‐finding and management services to PLHIV, PWID and other high‐risk groups (Appendix S1: description of the Flagship Project and activities). Services were provided by 19 local implementing NGOs. Public sector AIDS Centers provided care and treatment for cases identified.

Eligible index cases included PLHIV newly identified through Flagship's case‐finding, referred from public sector HIV programmes or found through lost‐to‐follow‐up tracing. Index cases were classified as newly found (diagnosed with HIV for the first time by Flagship), pre‐antiretroviral therapy (ART) (enrolled in care at an AIDS Center, but not yet on ART), newly started on ART (initiating ART for the first time after enrolling in Flagship's case management programme) or lost‐to‐follow‐up (not enrolled in care at an AIDS Center within the previous six months or who were enrolled but missed scheduled HIV care visits). Programme recipients included PWID, men who have sex with men (MSM), female sex workers (FSW), other PLHIV and their sex/ injecting partners. Individuals were eligible to receive Flagship's testing services if they were ≥18 years old and had not tested for HIV in the preceding six months.

Clients recruited to the programme were screened for eligibility, and eligible individuals provided basic demographic data (e.g. age, sex) via a paper‐based intake form administered by programme staff. Data on demographics, use of injection drugs, engagement in commercial/transactional sex, receipt of medication‐assisted therapy and history of migration were based on self‐report. Staff entered de‐identified data from paper forms into an online system. Clients were tracked using a unique identifier code, and no personal identifiers (e.g. name, phone number) were included in the database. Data were collected as a part of routine service delivery, and the Population Services International (PSI) Research Ethics Board granted a non‐research determination for this analysis. Flagship clients provided verbal consent for HIV testing and the collection of health‐related data.

### 2.2 Intervention design and procedures

Prior to APN's rollout Flagship offered passive partner testing services in which eligible PLHIV were counselled on the importance of disclosure and were offered recruitment coupons to distribute to their sexual/injecting partners (e.g. “coupon‐based recruitment” 14). These coupons included information about accessing testing services. Index cases who declined coupons were encouraged to refer partners directly, but no active follow‐up assistance was provided during this “pre‐APN period.”

Under APN, consenting index cases provided a listing of partners, and were offered APN through three mechanisms: (1) dual‐referral, when a trained peer navigator accompanied PLHIV to disclose their status to partners; (2) contract referral when PLHIV signed a contract with a peer navigator, under which they were given 30 days to disclose their status to partners and recommend they undergo voluntary testing. Partners not accessing testing within 30 days were contacted directly by the peer navigator who recommended they undergo testing without disclosing information about the PLHIV; or (3) provider referral, when PLHIV consent for the peer navigator to confidentially contact their partners directly to offer voluntary testing. Index clients could also choose coupon‐based recruitment instead of APN.

Implementing NGOs were trained on APN using the WHO and U.S. Centers for Disease Control and Prevention tools 8, 15, adapted for the local context. After confirmation of HIV status, clients were offered case management services from Flagship's peer navigators, and were linked to public sector care and treatment. After enrolment in case management, these individuals were also asked to serve as index clients for an additional round of partner testing.

For the purposes of this analysis, the APN period began one month after the APN training in each country to allow for a wash‐out period during intervention scale‐up. These data were compared to a similar time period in the calendar year preceding APN implementation. In Kazakhstan and the Kyrgyz Republic, the pre‐APN period went from October 2016 to September 2017, while the APN period went from October 2017 to September 2018. Because the APN scale‐up occurred later in Tajikistan, the pre‐APN period covered February 2017 to September 2017, and the APN period went from February 2018 to September 2018.

### 2.3 HIV testing

HIV testing was conducted according to national algorithms, and occurred in national AIDS Centers or community‐based testing. Clients testing positive on a rapid diagnostic test (RDT) were escorted to an AIDS Center for confirmatory testing or confirmed in the community. Clients diagnosed with HIV were traced in the national treatment database by AIDS Center staff to identify those already enrolled in care. Identifiable data used for tracing were not entered into the project database.

### 2.4 Statistical analysis

Data were extracted from Flagship's routine monitoring system. To understand differences by country and period, we compared the characteristics of index cases and partners recruited across countries and between the pre‐APN and APN periods, using descriptive statistics. To examine programme outcomes, we also compared the number of partners tested, the number/proportion of new testers, the number/proportion of women tested, the age of recruits, the number of new HIV cases found, and the positivity rate (the proportion of partners tested who were positive on their first HIV test) across countries and between the two periods. Binary variables were compared using Pearson's chi‐squared test, and t‐tests were used for continuous variables.

We described the proportion of index clients choosing each of the APN options using additional monitoring data from a subset of index cases. Finally, to determine whether the pre‐APN and APN periods were comparable in terms of index clients recruited, we used logistic regression to examine demographic and clinical factors (age, sex, country, use of injection drugs, treatment status, marital status, employment and disclosure of HIV status) associated with being an index case in the APN versus the pre‐APN period. Univariate models were fitted, and demographic factors were added based on univariate significance of <0.10. Model fit was compared using the Bayesian information criterion, and variance inflation factors were evaluated for multicollinearity. All analyses were performed using STATA 13.0 (Stata Corporation, College Station, TX, USA).

## 3 Results

### 3.1 Description of index cases across countries

Altogether 2675 PLHIV index cases were recruited during the APN period (Table 1). Of these, 1090 (41%) were recruited from Kazakhstan, 956 (36%) from the Kyrgyz Republic and 629 (24%) from Tajikistan. Most index cases were male (1669, 62%), with a median age of 37 years (interquartile range (IQR): 32 to 44). Nearly a third of index cases reported being married (30%), though this varied by country. In Kazakhstan, only 15% of index cases reported being married, versus 28% in the Kyrgyz Republic and 60% in Tajikistan (*p* < 0.001).

**Table 1:** Demographic and clinical characteristics of people living with HIV serving as index cases before and after the introduction of assisted partner testing in Kazakhstan, the Kyrgyz Republic and Tajikistan

Variable	Total (N = 7093)			Kazakhstan (N = 2581)			Kyrgyz Republic (N = 2372)			Tajikistan (N = 2140)		
	Pre‐APN (n = 4418, 62.3)	APN (n = 2675, 37.7%)	*p*‐value	Pre‐APN (n = 1491)	APN (n = 1090)	*p*‐value	Pre‐APN (n = 1416)	APN (n = 956)	*p*‐value	Pre‐APN (n = 1511)	APN (n = 629)	*p*‐value
	N (%)	N (%)		N (%)	N (%)		N (%)	N (%)		N (%)	N (%)	
Female	1850 (41.9)	1006 (37.6)	<0.001	568 (38.1)	406 (37.3)	0.661	622 (43.9)	367 (38.4)	0.007	660 (43.7)	233 (37.0)	0.005
Age (median, interquartile range)	38 (33 to 45)	37 (32 to 44)	0.001	38 (33 to 43)	37 (32 to 43)	0.002	39 (33 to 45)	39 (32.5 to 45)	0.764	39 (32 to 45)	36 (30 to 44)	<0.001
Marital status												
Currently married	1668 (38.7)	798 (30.0)	<0.001	262 (18.6)	157 (14.5)	0.035	515 (37.1)	267 (28.2)	<0.001	891 (59.0)	374 (59.6)	0.010
Cohabitating	652 (15.1)	468 (17.6)		417 (29.6)	325 (30.0)		189 (13.6)	115 (12.1)		46 (3.0)	28 (4.5)	
Unmarried/not cohabitating	1178 (27.3)	857 (32.2)		542 (38.4)	468 (43.2)		358 (25.8)	260 (27.5)		278 (18.4)	129 (20.5)	
Divorced	539 (12.5)	425 (16.0)		147 (10.4)	101 (9.3)		255 (18.4)	265 (28.0)		137 (9.1)	59 (9.4)	
Widow	273 (6.3)	110 (4.1)		43 (3.1)	32 (3.0)		71 (5.1)	40 (4.2)		159 (10.5)	38 (6.1)	
Project entry												
Newly found	395 (9.0)	560 (20.9)	<0.001	70 (4.7)	108 (9.9)	<0.001	157 (11.2)	224 (23.4)	<0.001	168 (11.1)	228 (36.3)	<0.001
Lost‐to‐follow‐up	849 (19.3)	361 (13.5)		292 (19.6)	136 (12.5)		253 (18.0)	118 (12.3)		304 (20.1)	107 (17.0)	
Pre‐ART care	3165 (71.8)	1754 (65.6)		1129 (75.7)	846 (77.6)		997 (70.9)	614 (64.2)		1039 (68.8)	294 (46.7)	
Population												
People who inject drugs	1598 (36.2)	845 (31.6)	<0.001	712 (47.8)	485 (44.5)	0.398	392 (27.7)	216 (22.6)	<0.001	503 (33.3)	144 (22.9)	<0.001
Men who have sex with men	46 (1.0)	76 (2.8)		34 (2.3)	26 (2.4)		11 (0.8)	32 (3.4)		1 (0.1)	18 (2.9)	
Sex workers	31 (0.7)	6 (0.2)		4 (0.3)	2 (0.2)		5 (0.4)	3 (0.3)		22 (1.5)	1 (0.2)	
Other	2734 (62.0)	1748 (65.4)		741 (49.7)	577 (52.9)		1008 (71.2)	705 (73.7)		985 (65.2)	466 (74.1)	
Employment status												
Regular job	596 (13.8)	375 (14.1)	<0.001	321 (22.6)	224 (20.7)	0.007	161 (11.6)	121 (12.7)	0.003	114 (7.5)	30 (4.8)	<0.001
Temporary job	993 (23.0)	746 (28.0)		337 (23.8)	317 (29.2)		376 (27.1)	275 (28.9)		280 (18.5)	154 (24.6)	
Unemployed	2529 (58.6)	1430 (53.7)		693 (48.8)	511 (47.1)		786 (56.6)	481 (50.5)		1050 (69.5)	438 (69.9)	
Student	19 (0.4)	19 (0.7)		8 (0.6)	5 (0.5)		8 (0.6)	13 (1.4)		3 (0.2)	1 (0.2)	
Other	181 (4.2)	94 (3.5)		60 (4.2)	27 (2.5)		57 (4.1)	63 (6.6)		64 (4.2)	4 (0.6)	
Used coupon‐based recruitment^a^	1155 (26.1)	693 (25.9)	0.820	673 (45.1)	413 (37.9)	<0.001	223 (15.8)	178 (18.6)	0.067	259 (17.1)	102 (16.2)	0.603
Has not disclosed status to anyone	1111 (25.7)	989 (37.1)	<0.001	229 (16.2)	260 (24.0)	<0.001	528 (37.9)	540 (56.7)	<0.001	354 (23.4)	189 (30.1)	0.001
ART status												
Never on ART	1133 (25.7)	826 (30.9)	<0.001	621 (41.7)	327 (30.0)	<0.001	370 (26.1)	348 (36.4)	<0.001	142 (9.4)	151 (24.0)	<0.001
Newly started	1087 (24.6)	1165 (43.6)		368 (24.7)	513 (47.1)		387 (27.3)	364 (38.1)		332 (22.0)	288 (45.8)	
Reinitiated	1792 (40.6)	489 (18.3)		415 (27.8)	156 (14.3)		534 (37.7)	210 (22.0)		843 (55.8)	123 (19.6)	
Already on ART	24 (0.5)	0 (0.0)		2 (0.1)	0 (0.0)		22 (1.6)	0 (0.0)		0 (0.0)	0 (0.0)	
Previously on ART	382 (8.7)	195 (7.3)		85 (5.7)	94 (8.6)		103 (7.3)	34 (3.6)		194 (12.8)	67 (10.7)	
MAT participant^b^	135 (13.4)	20 (5.8)	<0.001	–	–	–	63 (17.4)	17 (8.2)	0.002	69 (13.7)	3 (2.2)	<0.001

APN, assisted partner notification; ART, antiretroviral therapy; MAT, medication‐assisted therapy.

^a^Coupon‐based recruitment was a passive partner recruitment approach based on respondent driven sampling. Index cases choosing this option were provided with coupons containing HIV testing information to distribute to their sexual and injecting partners. This is relative to all other passive approaches (during the pre‐APN period) or all other passive and active approaches (during the APN period); ^b^MAT data were only available for 1,354 index cases (1,007 during the pre‐APN period and 347 during the APN period).

Across countries, most index cases from the APN period were recruited from pre‐ART care services (66%), were newly diagnosed through HIV case‐finding activities (21%) or through lost‐to‐follow‐up tracing (13.5%). Most index cases (65%) were PLHIV not identifying as a KP, while 32% identified as PWID. Few index cases reported being MSM (3%) or FSW (0.2%). Most reported being unemployed (54%), though this was significantly higher in Tajikistan (70%) than in the Kyrgyz Republic (51%) or Kazakhstan (47%, *p *< 0.001).

### 3.2 Comparison of index cases during the pre‐APN and APN periods

Altogether 4418 index cases were recruited during the pre‐APN period, approximately 1.7 times as many recruited during the APN period (Table 1). Pre‐APN index cases were significantly more likely than their APN counterparts to be female (42% vs. 38%, *p *< 0.001), to be married (39% vs. 30%, *p *< 0.001), to report injecting behaviour (36% vs. 32%, *p *< 0.001) and to be unemployed (59% vs. 54%, *p *< 0.001). Pre‐APN index cases were also significantly less likely to have been newly found through Flagship's case‐finding activities (9% vs. 21%, *p *< 0.001), to have not disclosed their HIV status to anyone (26% vs. 37%, *p *< 0.001) or to have been newly started on ART (33% vs. 63%, *p *< 0.001).

During both periods, only about a quarter (26%) of index cases chose to use coupon‐based recruitment. Women were more likely than men to choose coupon‐based recruitment (30% vs. 23%, *p *< 0.001), as were index cases from Kazakhstan (42% vs. 17% in the Kyrgyz Republic and Tajikistan, *p *< 0.001). Index cases newly started on ART were also more likely to choose coupon‐based recruitment (32%) than clients who were already on ART (13%), who had reinitiated ART (22%) or who had previously been on ART (23%, *p *< 0.001). PWID were less likely than other index cases to choose coupon‐based recruitment (18% vs. 31%, *p *< 0.001), as were individuals who were married (23% vs. 28%, *p *< 0.001).

Separate monitoring data from 813 APN index cases found a diversity in preferences between contract, provider and dual‐referral options. Across countries contract referral was most popular (333, 41%), followed by provider‐led referral (309, 38%) and dual‐referral (171, 21%). After adjusting for age, sex and country of residence, APN index cases had increased odds of being newly found PLHIV (adjusted odds ratio (aOR): 2.22, 95% CI: 1.75 to 2.83) or enrolled in pre‐ART care (aOR: 1.30, 95% CI: 1.03 to 1.37, relative to being traced from lost‐to‐follow‐up), and to have no one know their HIV status (aOR: 1.55, 95% CI: 1.33 to 1.79, relative to having disclosed to anyone) compared to the pre‐APN period. After adjusting for these factors, index cases in the APN period were less likely than their pre‐APN counterparts to choose coupon‐based recruitment (aOR: 0.73, 95% CI: 0.65 to 0.83) or to report the use of injection drugs (aOR: 0.56, 95% CI: 0.49 to 0.63).

### 3.3 Partners’ characteristics across countries

During the APN period, 3735 partners were recruited for testing (Table 2). Most partners were recruited from the Kyrgyz Republic (1667, 45%), followed by Kazakhstan (1068, 29%) and Tajikistan (1000, 27%). Females made up the majority of partners in the Kyrgyz Republic (54%) and Tajikistan (58%), but not in Kazakhstan (46%, *p* < 0.001). Partners had a median age of 36 years (IQR: 29 to 42), and few reported ever testing for HIV prior to being recruited (11% in Tajikistan and 16% in the Kyrgyz Republic, *p* = 0.003). Among those recruited through coupons, most partners reported having had sex with their recruiter (98%), though a small proportion (4%) reported needle sharing.

**Table 2:** Demographic characteristics of sex and injecting partners identified in the pre‐assisted partner testing and post‐assisted partner testing periods in Kazakhstan, the Kyrgyz Republic and Tajikistan

Variable	Total (N = 5980)			Kazakhstan (N = 2401)			Kyrgyz Republic (N = 2072)			Tajikistan (N = 1507)		
	Pre‐APN (N = 2245, 37.5%)	APN (N = 3735, 62.5%)	*p*‐value	Pre‐APN (N = 1333, 55.5%)	APN (N = 1068, 44.5%)	*p*‐value	Pre‐APN (N = 405, 19.6%)	APN (N = 1667, 80.5%)	*p*‐value	Pre‐APN (N = 507, 33.6%)	APN (N = 1000, 66.4%)	*p*‐value
	N (%)	N (%)		N (%)	N (%)		N (%)	N (%)		N (%)	N (%)	
Female	1129 (50.3)	1960 (52.5)	0.101	618 (46.4)	478 (44.8)	0.433	258 (63.7)	859 (51.5)	<0.001	253 (49.9)	623 (62.3)	<0.001
Age (median, interquartile range	37 (31 to 44)	35 (29 to 42)	<0.001	37 (31 to 43)	36 (30 to 42)	0.018	38 (31 to 45)	36 (30 to 43)	0.010	37 (30 to 44)	34 (28 to 40)	<0.001
Tested for HIV previously^a^	204 (22.4)	193 (12.6)	<0.001	–	–	–	128 (31.8)	85 (16.1)	<0.001	76 (15.0)	108 (10.8)	0.019
Shared needles with recruiter^b^	28 (1.5)	79 (4.3)	<0.001	2 (0.2)	3 (0.3)	0.465	20 (5.1)	13 (2.5)	0.035	6 (2.8)	63 (26.8)	<0.001
Had sex with recruiter^c^	1927 (99.7)	1791 (97.8)	<0.001	1311 (99.9)	1066 (99.8)	0.448	400 (99.0)	524 (99.2)	0.703	216 (100)	201 (85.5)	<0.001
Migration experience^d^	224 (44.2)	329 (32.9)	<0.001	–	–	–	–	–	–	224 (44.2)	329 (32.9)	<0.001
Positive HIV test	161 (7.2)	322 (8.6)	0.048	58 (4.4)	65 (6.1)	0.058	32 (7.9)	105 (6.3)	0.247	71 (14.0)	152 (15.2)	0.537
Year traced												
2016	202 (9.0)	0 (0.0)	<0.001	186 (14.0)	0 (0.0)		16 (4.0)	0 (0.0)	<0.001	0 (0.0)	0 (0.0)	<0.001
						<0.001						
2017	2043 (91.0)	538 (14.4)		1147 (86.1)	324 (30.3)		389 (96.1)	214 (12.8)		507 (100)	0 (0.0)	
2018	0 (0.0)	3197 (85.6)		0 (0.0)	744 (69.7)		0 (0.0)	1453 (87.2)		0 (0.0)	1000 (100)	
Where HIV tested												
RDT at external site	109 (4.9)	3 (0.08)	<0.001	0 (0.0)	0 (0.0)	<0.001	13 (3.2)	0 (0.0)	<0.001	96 (18.9)	3 (0.3)	<0.001
RDT at implementing NGO	1514 (67.4)	3723 (99.7)		712 (53.4)	1061 (99.3)		391 (96.5)	1665 (100)		411 (81.1)	997 (99.7)	
ELISA at external site	622 (27.7)	7 (0.2)		621 (46.6)	7 (0.7)		1 (0.3)	0 (0.0)		0 (0.0)	0 (0.0)	

APN, assisted partner notification; NGO, non‐governmental organization; RDT, rapid diagnostic test.

^a^HIV testing data were not collected in Kazakhstan. Additionally, 1142 partners in the Kyrgyz Republic (2 in the pre‐APN period and 1140 in the APN period) were missing data on HIV testing history; ^b^2230 partners were missing data on needle sharing with their recruiter (324 pre‐APN and 1906 APN). By country, 24 partners in Kazakhstan (22 pre‐APN and 2 APN), 1150 in the Kyrgyz Republic (11 pre‐APN and 1139 APN) and 1056 from Tajikistan (291 pre‐APN and 765 APN) were missing data on needle sharing with their recruiter; ^c^2217 partners were missing data on having sex with their recruiter (313 pre‐APN and 1904 APN). By country, 21 partners from Kazakhstan (21 pre‐APN and 0 APN), 1140 partners from the Kyrgyz Republic (1 pre‐APN and 1139 APN) and 1056 from Tajikistan (291 pre‐APN and 765 APN) were missing data on having sex with their recruiter; ^d^migration data only collected in Tajikistan. Migration experience was defined as ever having lived or worked outside of the country. 1738 partners from the pre‐APN period and 2735 from the post‐APN period were missing data on migration experience.

### 3.4 Comparison of partners tested during the pre‐APN and APN periods

APN partners were slightly younger (median age 35 vs 37, *p* < 0.001) and less likely to have previously tested for HIV (13% vs. 22%, *p* < 0.001) than pre‐APN partners. Although we observed no significance overall or in Kazakhstan, partners recruited in the APN period were less likely to be female in the Kyrgyz Republic (52% vs. 64%, *p* < 0.001) and more likely to be female in Tajikistan (62% vs. 50%, *p* < 0.001) relative to the pre‐APN period.

**Figure 1:**
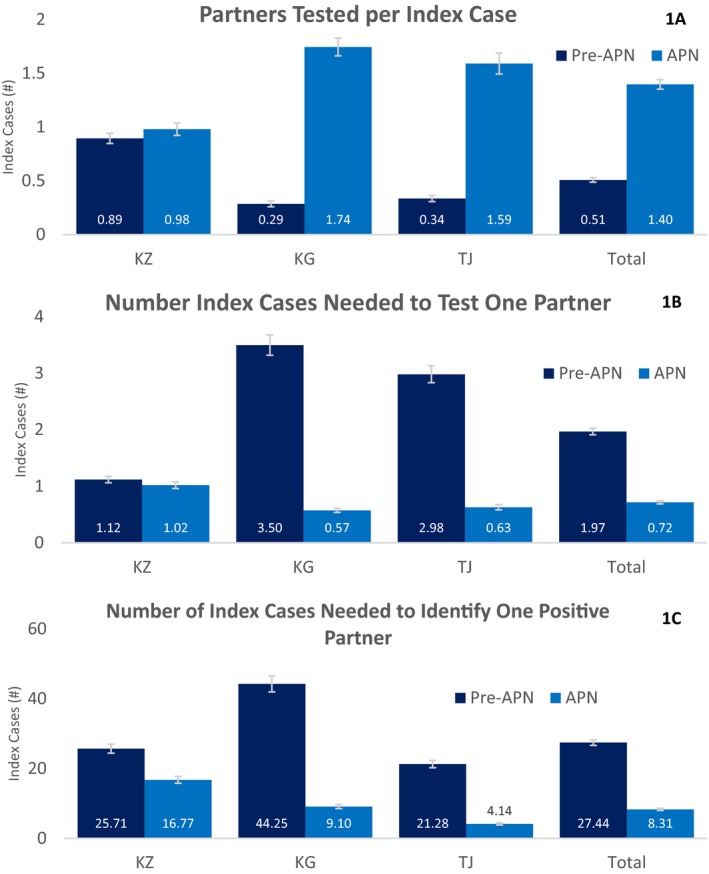
(A,B,C) Comparison of efficiency outcomes between the pre‐ and post‐assisted partner notification periods across Flagship Central Asia.

Altogether 8.6% of partners tested positive for HIV during the APN period, compared to 7.2% in the pre‐APN period (*p* = 0.048) (Table 2). The positivity rate increased non‐significantly between the pre‐APN and APN periods in Kazakhstan (4.4% vs. 6.1%, *p* = 0.058) and Tajikistan (14.0% vs. 15.2%, *p* = 0.537), but decreased in the Kyrgyz Republic (7.9% vs. 6.3%, *p* = 0.247). Though the positivity rate remained similar, the crude number of partners testing HIV positive increased between periods in Kazakhstan (58 vs. 65), the Kyrgyz Republic (32 vs. 105) and in Tajikistan (71 vs. 152) (Figure 1A,B,C).

Partners recruited per index case increased from 0.51 in the pre‐APN period to 1.4 in the APN period (*p* < 0.001, Figure 1A). This increase was larger in the Kyrgyz Republic (0.3 vs. 1.7, *p* < 0.001) and Tajikistan (0.3 vs. 1.6, *p* < 0.001) than in Kazakhstan (0.9 vs. 1.0, *p *= 0.109). Overall, the number of index cases needed to test one partner decreased from 2.0 in the pre‐APN period to 0.7 in the APN period (*p *< 0.001, Figure 1B), though again the magnitude of this change was larger in the Kyrgyz Republic (3.5 vs. 0.6, *p *< 0.001) and in Tajikistan (3.0 vs. 0.6, *p *< 0.001) than in Kazakhstan (1.1 vs. 1.0, *p *= 0.109). Finally, the number of index cases needed to identify one RDT‐positive partner fell from 27.4 in the pre‐APN period to 8.3 after APN implementation (*p *< 0.001, Figure 1C). The magnitude of this change was larger in the Kyrgyz Republic (44.3 vs. 9.1, *p* < 0.001) and in Tajikistan (21.3 vs. 4.1, *p* < 0.001) than in Kazakhstan (25.7 vs. 16.8, *p *= 0.020). Under APN, the positivity rate improved significantly among males (5.4% vs. 8.1%, *p *= 0.007), and among those who had previously tested for HIV (6.9% vs. 15.5%, *p *= 0.006).

## 4 Discussion

The Central Asia Flagship Project was able to recruit a large number of sexual and injecting partners and increase HIV case‐finding among partners using APN. While overall case‐finding improved substantially, positivity rates increased only slightly (7.2% vs. 8.6%), and were not significantly higher in the APN period than the pre‐APN period for any country. This may be partially attributable to passive, partner notification services provided by NGOs prior to the rollout of APN, and partner testing through routine epidemiological investigations conducted by the AIDS Centers for new HIV cases. However, under APN, the number of partners tested per index case recruited increased (0.5 to 1.4) and the number of index cases needed to find one positive partner decreased significantly (27.4 to 8.3).

Our findings varied substantially by country. We observed smaller changes in HIV case‐finding outcomes between the pre‐APN and APN periods in Kazakhstan relative to Tajikistan and the Kyrgyz Republic. Across both periods, index cases in Kazakhstan were more likely to be enrolled from pre‐ART care (76% and 78%) than index cases in either Tajikistan (69% and 47%) or the Kyrgyz Republic (71% and 64%). It is possible that these and other differences, including the proportion of index cases who were newly found PLHIV or who had not disclosed their HIV status to anyone yet, account for the greater impact of APN on outcomes in Tajikistan and the Kyrgyz Republic. However, without being able to link index cases with their partners in our data set, this cannot be definitively determined, and future research should explore this further.

Previous research has identified important barriers to partner notification, including concerns around privacy/confidentiality 8, 16, 17, 18. Our experiences suggest APN services were acceptable to PLHIV in Central Asia, including a large number of PWID living with HIV, but more work is needed to understand barriers to APN uptake in this population. While the evidence suggests that violence or harms are also rarely associated with APN 9, 10, 11, 12, 16, 17, this is an important concern for these programmes. Even with the low risk of violence, Flagship has added intimate partner violence screening for index cases and suspends APN efforts in partnerships where violence is reported. The project did not collect data on APN‐related social harms during the period under analysis, but subsequent data from the Kyrgyz Republic did not find any reports of harms associated with APN. Future work should more rigorously evaluate the safety and acceptability of APN in Central Asia, especially among PWID and women, who face additional legal and social vulnerabilities.

Notably, Flagship's APN services were successful in reaching high‐risk women with HIV testing. Altogether, 52% of partners tested through APN were women, and the proportion testing positive was similar to men (9.1% vs. 8.1%). Identifying and testing women at risk for HIV can be challenging in epidemics concentrated among PWID, where women face multiple and intersecting risks, including high levels of stigma/discrimination, lack of access to testing and HIV‐related prevention and care, and transmission risks from sexual partner(s) who inject, exchange of sex for drugs and risks associated with shared injecting equipment 7, 18, 20, 21. PWID‐focused HIV case‐finding interventions may also miss non‐injecting female sexual partners of male PWID, especially those who are not spouses. This is important in the Central Asian epidemics where additional attention is needed to support the diagnosis of non‐injecting sexual partners. Adding APN services to PWID‐focused case‐finding was a feasible strategy to find and test female partners of male PWID. However, when non‐KP individuals are found by KP‐focused organizations, special attention should be devoted to linkage to care and post‐test supportive services that meet the needs of these partners.

While our experiences suggest that scaling APN services alongside existing PWID HIV case‐finding programmes was feasible, concerted efforts were needed to ensure collaboration across multiple stakeholders. Strong relationships with the public sector AIDS Centers, including the ability to share data, were vital. This was especially important given that most index cases were recruited from public sector pre‐ART care or lost‐to‐follow‐up tracing, rather than Flagship's own HIV case‐finding. The relationship between the implementing NGOs and the target population were also crucial in reassuring index cases that their identity and HIV status would be kept confidential, and NGOs with less experience and/or trust in the communities found implementation of the APN intervention to be more difficult. Flagship continues to provide supportive supervision and ongoing training to gain further buy‐in for the intervention among NGOs, to improve relations with AIDS Centers and to allow highly successful NGOs to share experiences and best practices with organizations experiencing implementation challenges.

Future efforts should ensure programmes have systems that link index case data to that of their recruited partners, capture the proportion of PLHIV agreeing to serve as index cases, and the type of APN chosen. This would enable evaluations of which strategies are working best, what types of notifications are preferred, and allow additional research – including cost‐effectiveness studies – to be embedded within routine programming. Similar monitoring systems have been successfully developed for APN services in Kenya 21. Finally, public sector AIDS Centers and other partners should be encouraged to recognize the value of involving KP‐focused NGOs in conducting APN among PWID and other KPs because of their unique position in communities, and their ability to effectively provide services to sexual and injecting partners (including non‐spousal partners and those who do not identify as KP).

This analysis is subject to important limitations. First, the data used for this analysis were collected during routine monitoring system under programmatic conditions. The system was not designed to link index cases to their partners, and we were unable to assess whether differences in index case characteristics may have accounted for improved programme outcomes between periods, rather than the intervention or to assess factors behind country‐level heterogeneity. We were also unable to estimate the proportion of PLHIV who consented to APN, or the proportion of index cases who successfully recruited a partner, both of which seriously limit our ability to assess APN acceptability and effectiveness. Additionally, data were subject to a high degree of missingness for some variables, such as migration status. Other variables which might have been important in predicting HIV status among partners, including HIV‐related risk behaviours, were not collected as a part of routine monitoring and could not be included in the analysis.

Furthermore, information about risk behaviours (including use of injection drugs), ART status and previous HIV testing were based on self‐report and are subject to social desirability bias. It is possible that some index cases did not disclose commercial sex work or use of injection drugs and were misclassified as “other PLHIV.” Additionally, partners were not categorized as PWID/MSM/FSW/other. Finally, this analysis was a simple pre/post design, and passive partner notification services were provided by NGOs prior to the launch of APN. Future studies could consider a randomized designed in order to assess the effectiveness of APN in this population and setting. Despite these limitations, this analysis is one of the first contributions to the literature describing the implementation of APN among PWID and other hard‐to‐reach populations in Central Asia.

## 5 Conclusions

APN services were feasible in Central Asia, and were able to be implemented alongside other HIV case‐finding/management services. Though positivity rates varied considerably across countries, the addition of APN to a primarily PWID‐focused programme resulted in significant increases in the number of partners recruited per index case, and significant reductions in the number of index cases needed to find a new HIV‐positive partner. Focusing additional resources on APN, using good practice tools and methods, may be a feasible way to improve HIV case‐finding among hard‐to‐reach populations.

## Competing interests

The authors have no competing interests to declare.

## Authors’ contributions

KL performed the data analysis and led the manuscript writing. MK cleaned the data set and contributed to the analysis and manuscript writing. OS provided strategic direction for the analysis and contributed to manuscript writing. AR, DS and FI provided insights into programmatic lessons learned and assisted in manuscript writing and editing. RG and NH contributed to manuscript writing and provided technical inputs.

## Supporting information


**Appendix S1.** Description of the Flagship project and activities.
