# Supplementary material for: Implementation experiences and insights from the scale‐up of an HIV assisted partner notification intervention in Central Asia
Source: J Int AIDS Soc. 2019 Jul 19;22(Suppl Suppl 3):e25313. doi: 10.1002/jia2.25313 (PMC6639697; doi:10.1002/jia2.25313)
Supplement: Supplementary file 1 — Appendix S1. Description of the Flagship project and activities. [file JIA2-22-e25313-s001.docx]

**Supplemental Text 1: Description of the Flagship Project and Activities**

In Kazakhstan the Flagship Project is implemented in two sub-national units (SNUs): Eastern Kazakhstan region and the bordering Pavlodar region. In both sites there is a government-run Regional AIDS Centers, as well as three city AIDS Centers (two in Pavlodar region and one in Semei city, East Kazakhstan region). Registered PLHIV receive all HIV testing and treatment services in AIDS Centers. Other medical facilities do not provide HIV treatment. While HIV rapid-testing and ELISA testing are available in primary care facilities and hospitals, only government AIDS Centers can do confirmatory testing.

In the Kyrgyz Republic the Flagship Project is implemented in four SNUs: Bishkek city, Chui region, Osh city and Osh region. These territories have the highest HIV burden in the country and are also the geographies where the majority of key populations and PLHIV are located. The Kyrgyzstan National AIDS Program is managed by the Republican AIDS Center under the Ministry of Health. Each of the country’s 7 main regions has a Regional AIDS Center which provide confirmatory testing (ELISA), viral load and CD4 count testing, ART initiation, and counselling services. HIV treatment and care services have been further decentralized, with doctors from local primary healthcare facilities (Family Medicine Centers) being given training and rights to prescribe ART and conduct monitoring of adherence.

In Tajikistan the Flagship Project is implemented in three SNUs: Dushanbe city, Districts of the Republican Subordination (DRS) and Sughd Oblast. The Tajikistan National AIDS Program is managed by the Republican AIDS Center under the Ministry of Health and is located in Dushanbe city. There is also a city AIDS center in Dushanbe city. In Sughd Oblast there is a government-run Regional AIDS Center, as well as five city AIDS Centers (Khudjand, Gafurov, Kanibadam, Isfara, Istaravshan, and Penjikent). In DRS there are three city AIDS Centers (Hissar, Tursunzade, Vakhdat). Registered PLHIV receive all HIV testing and treatment services in AIDS Centers. Other medical facilities do not provide HIV treatment. Confirmatory testing can be provided at both Republican and Regional AIDS Centers.

Across all three countries HIV case finding is primarily carried out by local non-governmental community-based organizations (CBOs). The CBOs are typically staffed by people living with HIV and/or people who use drugs, along with social workers or other volunteers. The Flagship project trains and supports these CBOs to conduct HIV case finding using various methodologies, including coupon distribution through a respondent driven sampling approach, as well as on-going case-finding that focus on districts or locations with high suspected populations of people vulnerable to HIV. Where permitted, the community based HIV case finding includes rapid tests administered by outreach workers. The CBOs work very closely with the local AIDS Centers, to ensure cases found are effectively linked to care and treatment, frequently sharing data to enable tracking of clients across the HIV care cascade. In most cases, the local AIDS Centers also cooperate with the CBOs to locate PLHIV who have been lost to follow up, to relink them to care and treatment. Local AIDS Centers typically carry out ‘passive’ HIV case finding, meaning walk-in clients are offered free, anonymous HIV testing. On occasion, local AIDS Centers will also conduct periodic active HIV case-finding activities, for example to mark World AIDS Day.

CBO staff across all three countries provide counseling and case-management services to promote treatment initiation, retention in care, and partner discloser, including options for assisted partner notification. Peer navigators at the CBOs provide case management services for clients during the first 6 months following HIV diagnosis. During this time peer navigators work to build rapport with their PLHIV clients, ultimately offering them assistance with disclosure of their HIV status to partners and referrals to HIV testing. Various options—including assisted and unassisted approaches—are offered to clients, and the client chooses the most appropriate approach for each listed partner.
